# Determination of the Mineral Composition and Toxic Element Contents of Propolis by Near Infrared Spectroscopy

**DOI:** 10.3390/s151127854

**Published:** 2015-11-03

**Authors:** M. Inmaculada González-Martín, Olga Escuredo, Isabel Revilla, Ana M. Vivar-Quintana, M. Carmen Coello, Carlos Palacios Riocerezo, Guillermo Wells Moncada

**Affiliations:** 1 Departamento de Química Analítica, Nutrición y Bromatología. Facultad de Ciencias Químicas, Universidad C/ Plaza de los Caidos s/n. Salamanca 37008, Spain; 2 Departamento BiologíaVegetal y Ciencias del Suelo. Facultad de Ciencias, Universidad de Vigo, As Lagoas, Ourense 32004, Spain; E-Mails: oescuredo@uvigo.es (O.E.); mcoello@uvigo.es (M.C.C.); 3 Área de Tecnología de los Alimentos, Escuela Politécnica Superior de Zamora, Avda Requejo 33, Zamora 49022, Spain; E-Mails: irevilla@usal.es (I.R.); avivar@usal.es (A.M.V.-Q.); 4 Área de Producción Animal, Facultad de Ciencias Agrarias y Ambientales, Universidad. Avda, Filiberto Villalobos, Salamanca 119-129, Spain; E-Mail: carlospalacios@colvet.es; 5 Departamento de Producción Animal, Facultad de Agronomía, Universidad de Concepción, Avda. Vicente Méndez, 595 Chillán, Chile; E-Mail: guillermo.wells.moncada@gmail.com

**Keywords:** propolis, mineral composition, lead, near-infrared spectroscopy, determination, cross-validation

## Abstract

The potential of near infrared spectroscopy (NIR) with remote reflectance fiber-optic probes for determining the mineral composition of propolis was evaluated. This technology allows direct measurements without prior sample treatment. Ninety one samples of propolis were collected in Chile (Bio-Bio region) and Spain (Castilla-León and Galicia regions). The minerals measured were aluminum, calcium, iron, potassium, magnesium, phosphorus, and some potentially toxic trace elements such as zinc, chromium, nickel, copper and lead. The modified partial least squares (MPLS) regression method was used to develop the NIR calibration model. The determination coefficient (R^2^) and root mean square error of prediction (RMSEP) obtained for aluminum (0.79, 53), calcium (0.83, 94), iron (0.69, 134) potassium (0.95, 117), magnesium (0.70, 99), phosphorus (0.94, 24) zinc (0.87, 10) chromium (0.48, 0.6) nickel (0.52, 0.7) copper (0.64, 0.9) and lead (0.70, 2) in ppm. The results demonstrated that the capacity for prediction can be considered good for wide ranges of potassium, phosphorus and zinc concentrations, and acceptable for aluminum, calcium, magnesium, iron and lead. This indicated that the NIR method is comparable to chemical methods. The method is of interest in the rapid prediction of potentially toxic elements in propolis before consumption.

## 1. Introduction

Propolis are resinous substances collected from the buds and wounds of plants and transformed by honeybees. They use exuded resins as well actively secreted substances by plants that include lipophilic materials on leaves and leaf buds, gums, lattices, *etc.* Propolis is used in hives to reinforce the structural integrity of the hive, to seal entrances in wintertime, to reduce vibrations inside the hive, and also as an antiseptic agent. Propolis has different sensorial and physico-chemical properties but most propolis share considerable similarity in their general chemical composition: 50% resin, 30% wax, 10% volatile oils, 5% pollen and 5% other organic compounds [1,2]. The composition of propolis is very complex and varied depending on the phytogeographic diversity of the collection area and the season [2,3]. Propolis is a natural source of antioxidants, which protect oils and serum lipoprotein oxidation, highlighting its effects on antibody production and strengthening the immune system [4]. The presence of phenolic compounds, terpenes, steroids and amino acids in propolis has been studied extensively [5,6,7,8,9]. However, there is less information on the content of trace elements in propolis, especially the possible presence of toxic minerals, which can significantly affect its nutritional properties. Trace elements justify many virtues of propolis, as participating in metabolism, vitamin and fermentative processes, contributing to the healing of anemia, preventing arteriosclerosis and increasing the immune capacity of the body [4]. The mineral contents in propolis is used as a distinguishing feature of the geographical areas where it is produced [10,11], as an indicator of environmental pollution [12] and to develop reliable traceability methods [13]. The presence of toxic elements in propolis is associated with environmental pollution of anthropic origin around the apiaries through various sources, such as air, water, plants and soil. Some probable sources for cadmium and lead emissions are industrial sources [14,15,16,17]. Actually, some plant species are known and well characterized regarding their capacity to accumulate high levels of heavy metals in their biomass. They are classified as hyperaccumulators [18,19,20]. Thus, in regions where beekeeping is practiced for commercial purposes, the identification of bee plants with this characteristic is an important item to be evaluated, in order to assure that the product fully meets technical and sanitary specifications imposed by the regulatory agencies and the demanding consumer market in modern times [21]. Moreover, it is known that flavonoids tend to form stable complexes with metals such as iron, chromium, nickel, copper or lead [22]. This property makes these elements become one of the main pollutants of propolis.

The determination of the mineral composition of propolis is usually performed by ICP-mass analysis and also by Electrothermal Atomic Absorption Spectrometry (ET AAS) and UV-Vis spectrophotometry (UV-Vis) [23], neutron activation [10], electroanalytical methods [13], or in the case of lead content, using the Graphite Furnace Atomic Adsorption Spectrometry (GF AAS) method [24]. Thus, using flame atomic absorption spectrometry Formicki *et al.* [12] determined the levels of cadmium, iron, magnesium, nickel, lead and zinc in various bee products collected in Poland; Pierini *et al.* [13] used a electroanalytical method for quantification of lead in Argentine propolis; Serra-Bonvehí and Orantes-Bermejo [25] determined, among others, the levels of arsenic, cadmium, mercury and lead in samples of propolis collected in southern Spain by ICP-atomic emission spectrometry and Finger *et al*. [26], studied the content of cadmium, chromium and lead in Brazilian propolis from Paraná by electrothermal atomization and flame atomic absorption spectrometry after calcination in a muffle furnace. Moreover, Gong *et al*. [11] studied the relationships between the geographical origin and the content of calcium, aluminum, magnesium, potassium, iron, sodium, zinc, manganese, strontium, copper, chromium, nickel and toxic elements like arsenic, cadmium and lead determined by inductively coupled plasma atomic emission spectrometry after microwave digestion of Chinese propolis. However, there are few studies evaluating the potential of near infrared spectroscopy (NIR) for quantitative analysis of propolis. Visible/near infrared spectroscopy (Vis/NIRS) has been applied for the analysis of chrysin and galangin in Chinese propolis [27] and to explore its applicability for the determination of antioxidant properties [28].The detection of propolis falsification by the addition of flavonoid glycosides and tree latex has been carried out by Fourier Transform-NIR [29]. NIR has been also used to confirm the identity of isolated beeswax propolis [30]. Regarding the mineral characterization and to ensure the safety and quality of this product for marketing in local and international markets, this paper proposes a fast method for determining the mineral composition of propolis by using near infrared spectroscopy (NIR), because this analytical technique is rapid, non-destructive, and requires little or no sample preparation [31]. Therefore, in this paper, propolis samples from Chile and Galicia and Castilla-León (Spain) were analyzed with the aim of develop a rapid method of analysis to quantify some mineral and trace elements, using near-infrared spectroscopy with a remote reflectance fibre-optic probe applied directly to the sample.

## 2. Experimental

### 2.1. Propolis Samples

Propolis samples (*N* = 91) have been directly collected by beekeepers in Chile (52 samples: Bio-Bio region) and Spain (39 samples: Galicia and Castilla-León regions), from both organic and conventional farms (the samples are all from different farms, and are not duplicated). Samples were collected mostly with mesh and employing the scraping technique, keeping them frozen until used in the laboratory. For the NIR analysis, of all the 91 samples, 71 were employed for the denominated calibration set, and the other 20 were used for the external validation.

### 2.2. Chemical Analysis of the Mineral Composition

The mineral composition of propolis was determined using Inductively Coupled Plasma Optical Emission Spectrometry (ICP-OES) for aluminum, calcium, iron, potassium, magnesium, sodium and potassium, and Inductively Coupled Plasma Mass Spectrometry spectrometry (ICP-MS) for zinc, chromium, nickel, copper and lead. Propolis samples were crushed in the laboratory before their analysis with a grinder (Knifetec 1095 Sample Mill, Foss Tecator, Hohnaa, Sweden). Prior to analysis of mineral elements, the mineralization of the samples (0.2 g) in a microwave system (Ethos Sel Milestone, Ontario, ON, Canada) was performed and subsequently introduced into a high pressure capsule. In a first phase 5 mL of HNO_3_ was added and a power of 1000 watts was applied for 5 min. Once the sample was cool, another 5 mL of HNO_3_ and 1 mL of 30% H_2_O_2_ were added, applying a power of 1000 W for 10 min. The sample was cooled to room temperature, made up to 100 mL with distilled water and stored at 4 °C until analysis.

The ICP-OES determinations were carried out using Ultima 2 equipment (JobinYvon, Paris, NJ, USA), performing the calibration with certified standard solutions, ranging from 0.5 to 10 mg/kg. Detection limits were 0.1 mg/kg in solution. The ICP-MS determinations were performed on an Elan 6000 instrument (Perkin-Elmer, Wellesley, Massachusett, MA, USA). Do do this an internal standard (Sc, Y, Ho Ge 20 and 100 µg/kg) was added to an aliquot of previously prepared sample. The calibration was performed with certified standard solutions, also adding to the same internal standard for the calibration samples and adapted to a range of 10 to 200 µg/kg. Detection limits were 0.1 µg/kg in solution.

### 2.3. NIR Spectroscopy

**Figure 1:**
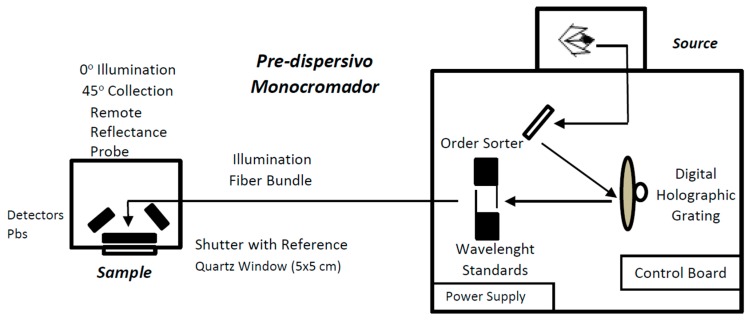
Schematic of the used NIRS equipment with fiber optic probe.

A Foss NIRSystem 5000 (Hillerod, Denmark) with a standard 1.5 m 210/210 bundle fibre-optic probe (Ref. n° R6539-A) was used. Figure 1 shows a diagram of the NIRS system, where the main components (optical system, sample module, fiber optic probe) can be observed. The reflectance detectors receive radiation from diffuse scattering of the sample. These detectors (four PbS elements) are positioned at 45° from the surface of the sample (to minimize the specular reflectance). The used spectral range is 1100–2000 nm, since above 2000 nm significant signal attenuation occurs because of the strong absorption of the -OH groups that may be present in the optical fiber. The probe employs a remote reflectance system and uses a ceramic plate as reference. The window is of quartz, with a 5 cm × 5 cm surface area. The measurement of the spectra was carried out using NIRS technology and a remote reflectance fibre-optic probe that was applied directly to samples of crushed propolis. The spectra were recorded at intervals of 2 nm, performing 32 scans for both the reference and samples. To minimise sampling errors, all the samples were analysed in triplicate.

### 2.4. Statistical Data Analysis

#### 2.4.1. Principal Component Analysis

Principal component analysis (PCA) was carried with the NIR spectral data from samples of the calibration set using WinISI II version 1.50 (Intrasoft International, LLC, Silver Spring, Maryland, MD, USA). This analysis transforms the original variables (wavelengths) into new axes called principal components, which are orthogonal, so that the data set presented on these axes are uncorrelated with each other. Spectra were pretreated by different techniques, including multiplicative scatter correction (MSC), standard normal variate (SNV), DT (Detrend) or SNV-DT [32]. These treatments allow minimization of the scattering effect, since mainly the shift of the maximum and the width changes of the spectra were considered.

#### 2.4.2. Modified Partial Least Squares Regression

The modified partial least squares (MPLS) regression method was used to obtain the NIR equations for the minerals quantified in propolis. The principal aim was generate models that allow the prediction of these components in the propolis matrix. Partial least squares (PLS) regression is similar to principal component regression (PCR), but uses both reference data and spectral information to form the factors useful for fitting purposes. To optimize the multivariate regression equations, the spectral scattering effects were taken into account with several mathematical treatments (MSC; SNV; DT or SNV-DT) [32]. MPLS is often more stable and accurate than the standard partial least squares algorithm (PLS). Calibrations were performed by modified partial least squares regression (MPLS) for each component, after removing the samples for spectral (criterion H) or chemical reasons (criterion T). The criterion H (Mahalanobis distance) explains the difference of the spectrum of an unknown sample from the mean spectrum of the set of samples. Samples with an H-value higher than 3 are considered as a different population and are eliminated. Furthermore, the risk of there being mistakes in the equations under practical conditions is very low or almost null when the standardised H statistic (Mahalanobis distance) is used during routine analysis of unknown samples. Using the *T* > 2.5 criterion, samples that were different from the population owing to the chemical criterion were removed from the set. In MPLS, the NIR residuals at each wavelength, obtained after each factor has been calculated, are standardized (dividing by the standard deviations of the residuals at each wavelength) before calculating the next factor [33]. In order to select the optimal number of factors and to avoid over fitting, cross-validation is recommended [34]. The calibration set is divided into several groups for the cross-validation. Each group is then validated using a calibration developed on the other samples. Validation errors generated are combined into a root mean square error of cross-validation RMSECV [35]. It has been reported that the RMSECV is the best single estimate for the prediction capability of the equation and that this statistic is similar to the average root mean square error of prediction (RMSEP) from 10 randomly-chosen prediction sets. In all cases, cross-validation was performed by splitting the population into six groups. Squared correlation coefficient was considered for the realization of models. The squared correlation coefficient for predicted versus measured compositions in cross-validation and the ratio of standard deviation (SD) to RMSECV of data set have been used. This ratio (of the SD to the RMSECV) is called the ratio of performance to deviation (RPD). This ratio is desired to be larger than 2 for a good calibration [36]. An RPD ratio less than 1.5 indicates poor predictions and the model cannot be used for further prediction. The statistics used to select the best equations were R^2^, determination coefficient; RMSEC, root mean square error of calibration; RMSECV, root mean square error of cross-validation [34].

## 3. Results and Discussion

### 3.1. Chemical Composition

The propolis samples had wide ranges in the mineral composition and high standard deviation values for the studied elements. Table 1 shows the mean concentration and the range of values of the mineral composition (aluminum, calcium, iron, potassium, magnesium, sodium, phosphorus, zinc, lead, chromium, nickel and copper) of all the propolis samples according to the geographical origin obtained by the chemical reference methods (ICP-OES and ICP-MS).

**Table 1:** Mineral composition of propolis studied according to geographic origin (mg/kg).

Constituent	Total (*N* = 91)		Chile (*N* = 52)		Galicia (*N* = 16)		Castilla-León (*N* = 23)	
	Mean	Range	Mean	Range	Mean	Range	Mean	Range
Al	275.2	43.0–833.9	354.9a	156.0–833.9	105.2b	43.0–193.7	213.2c	78.6–518.4
Ca	833.4	219.1–5173.0	910.4	274.0–5173.0	563.2	219.1–1176.1	847.3	416.7–2169.2
Fe	424.6	46.1–1538.0	536.6a	181.8–1538.0	245.8b	46.1–656.7	295.8b	104.5–874.0
K	978.6	267.0–4428.3	550.0a	267.0–1841.2	1522.1b	359.0–3182.1	1569.4b	685.9–4428.3
Mg	234.1	63.5–1398.0	261.8	75.1–1398.0	206.4	63.5–427.0	190.6	88.2–460.3
P	235.0	116.0–729.0	228.8a	118.1–402.0	307.5b	152.3–729.0	198.5a	116.0–327.7
Cr	3.7	0.8–48.9	3.1	1.4–5.5	2.7	0.8–7.5	5.7	2.3–48.9
Cu	1.8	Nd–33.4	1.6a	Nd–6.2	5.4b	Nd–33.4	2.8	Nd–7.2
Ni	1.5	Nd–29.9	1.2	Nd–9.7	1.4	0.5–4.0	2.4	0.6–29.9
Pb	5.8	Nd–73.9	2.6a	Nd–8.0	2.2a	Nd–6.0	15.5b	Nd–74.0
Zn	62.6	5.5–460.7	57.8	5.5–105.0	89.8	17.4–460.7	54.4	11.1–145.3

Statistical differences evaluated with Bonferroni test are marked at *p* < 0.05. Different letters indicate significant differences between groups. Nd: Not detected, below quantification level (0.01 mg/kg).

Also, a comparative study of the mineral composition of propolis from other geographical origins is shown in Table 2.

The possibility of correlation between all elements was investigated. Potassium, the most abundant mineral compound, was significantly (*p* < 0.01) and negatively correlated with aluminum (*r* = −0.29) and iron (*r* = −0.32), and positively correlated with phosphorus (*r* = 0.28), lead (*r* = 0.33) and copper (*p* < 0.05, *r* = 0.26). Iron was positively correlated (*p* < 0.01) with aluminum (*r* = 0.57), as observed for potassium, and also with calcium (*r* = 0.36), magnesium (*r* = 0.35) and zinc (*r* = 0.31). Magnesium showed a strong correlation with calcium (*r* = 0.97) and a significant correlation (*p* < 0.01) with phosphorus (*r* = 0.31). Finally, regarding the potentially toxic elements beside the significant correlation between lead and potassium, a strong correlation was found between chromium and nickel (*r* = 0.90).This result differs from previously reported by Finger *et al*. [26] who found a significant correlation between calcium and potassium and from those reported by Formicki *et al*. [12] who found a significant but negative correlation between iron and magnesium. These two works also found a positive correlation between cadmium and lead that they attributed to a common polluting source. However in this work the cadmium was not analyzed but a strong correlation between chromium and nickel was found, metals that were not analyzed in those works.

**Table 2:** Mineral composition of propolis samples of different geographic origin (mg/kg).

Constituent	South Spain [25] (*N* = 25)	Argentina [5] (*N* = 10)	Argentina [10] (*N* = 96)	China [11] (*N* = 32)	Brazil [26] (*N* = 42)
Al	308–582	–	–	426–1959	Nd–1840
Ca	1773–6683	39–4138	–	404–2637	Nd–4800
Fe	312–1270	101–1697	400–1945	310–2125	–
K	735–4790	101–1697	–	314v1894	410–5490
Mg	301–1405	1115–1031	–	135–1129	500–4650
P	171–611	–	–	–	–
Cr	0.3–3	Nd	0.6–3.7	Nd–12	Nd–19
Cu	2.1–4	Nd	–	Nd–15	Nd
Ni	0.6–3	Nd	–	Nd–3	–
Pb	0.07–4	–	–	4–55	Nd–160
Zn	163–1236	33-147	11–105	35–386	Nd–500
**Constituent**	**Poland [37,38]** **(*N* = 20)**	**Croatia [39]**	**Poland [12]** **(*N* = 80)**	**Turkey [40]**	
Al	–	–	–	–	
Ca	–	40–317	–	79–118	
Fe	28–101	14–251	101	–	
K	–	51–117	8.2	121–364	
Mg	137–823	10–46	–	–	
P	–	–	–	–	
Cr	–	0–1	–	–	
Cu	–	0.3–6	–	45–96	
Ni	2–10	0–0.3	9.8	–	
Pb	0.9–3	0.3–64	2.7	–	
Zn	18–71	8–933	71.5	176–676	

Mean value; [5] Lima *et al.*; [10] Cantarelli *et al.*; [11] Gong *et al.*; [12] Formicki *et al*.; [25] Serra-Bonvehí, and Orantes-Bermejo; [26] Finger *et al.*; [37] Roman *et al*.; [38] Roman *et al*.; [39] Cvek *et al.*; [40] Dogan *et al*.

### 3.2. NIR Calibration Equations

For the calibration equations, a set of 71 samples of propolis from different sources (Galicia, Castilla-León, Chile) was used. Initially, the principal component analysis (PCA) was performed. In all cases, the spectral variability explained above was 99%. Using both criteria (criterion H and T) six samples were deleted for the calibration of aluminum, 11 for the calcium, six for iron, 13 for potassium, seven for magnesium, five for phosphorus, 10 for chromium, 13 for copper, four for nickel, 12 for lead and seven for zinc. Calibrations were performed by modified partial least squares regression (MPLS), using the spectral data and chemical data matrix (obtained with ICP-OES, ICP-MS). The best of the different mathematical treatments, the concentration range, standard deviation and the calibration parameters for all elements are shown in Table 3.

**Table 3:** Statistical descriptors of calibration by NIR of the minerals.

Constituent	Math treatment	N	Mean	SD	Est. Min	Est. Max	RMSEC	R^2^	RMECV	RPD
Al	Standard MSC 1,4,4,1	65	257.4	123.9	0.0	629.2	56.5	0.79	78.8	1.6
Ca	None 1,4,4,1	60	509.4	245.9	0.0	1247.0	102.7	0.83	162.1	3.1
Fe	None 1,4,4,1	64	425.6	231.0	0.0	1118.6	129.3	0.69	147.3	1.6
K	Detrend only 2,4,4,1	58	772.8	559.6	0.0	2451.7	126.7	0.95	244.3	2.3
Mg	None 1,4,4,1	64	198.4	193.7	0.0	779.4	105.6	0.70	160.6	1.2
P	Standard MSC 1,4,4,1	66	236.7	103.9	0.0	548.4	26.0	0.94	40.4	2.6
Cr	SNV only2,4,4,1	61	2.9	0.8	0.5	5.3	0.6	0.48	0.8	1.0
Cu	Detrend only 2,10,10,1	58	1.2	1.6	0.0	5.8	0.9	0.64	1.2	1.3
Ni	None 0,0,1,1	67	1.2	1.0	0.0	4.2	0.7	0.52	1.0	1.0
Pb	None 1,4,4,1	59	3.5	3.7	0.0	14.6	2.0	0.70	3.3	1.1
Zn	SNV only 2,4,4,1	64	57.4	28.9	0.0	144.2	10.6	0.87	18.7	1.6

N, number of samples; SNV, standard normal variate; MSC, multiplicative scatter correction; SD, standard deviation; Est. Min: minimum value estimated by the model developed; Est Max: Maximum value estimated by the model developed; RMSEC, root mean square error of calibration; R^2^, determination coefficient; RMSECV, root mean square error of cross-validation; RPD, ratio of performance to deviation.

The quantification of the chemical elements using NIR technology is possible thanks to the distinct associations of these elements with the organic and inorganic material and with the water molecules, because of relative strong absorption of the overtones and combination modes of OH, sulphates and carbonate groups. Since 1981, there have been several reports on mineral elements in plants determined using NIR [41,42,43,44]; if NIRS can be used for determining mineral concentrations this is due to the association between minerals and organic functional groups or the organic matrix [45]. When NIR radiation is absorbed by molecules the energy is converted to molecular vibration energy. Molecules which are infrared (IR)-active are those which undergo a change in the dipole moment during transition, this means that bonds commonly found in biological systems such as C-H, O-H and N-H bonds are IR active. The prediction of trace elements by NIRS in agricultural products has been reported even less frequently and has always been used in the context of plants, only a few reports were found related to the use of NIRS for macro and trace minerals in both grasses and hay samples [46], in botanical fractions of semi-arid grassland [47] or legumes [48]. In the case of the propolis, the most likely association of minerals with organic matter is through flavonoids. Propolis presents a general composition of 40%–50% resins and balsams (that contain, in turn, 50% flavonoids and phenolic acids), 30%–40% wax, 10% volatile oils, 5% pollen and 5% minerals and other organic compounds. The chemical structures of flavonoids favour the formation of very stable complexes with heavy metals, making propolis a hyperaccumulator of these kinds of elements [18,19,20]. The correlation between the concentration and that measured at different wavelengths is given by the expression: *y* = *β*_0_ + *β*_1_ *X_λ_*_1_ + *β*_2_ *X_λ_*_2_ + *β*_3_ *X_λ_*_3_ +…+ *β*_n_ *X_λ__v_*, where *β* are the coefficients and *X_λ_*_1_, *X_λ_*_2_, *X_λ_*_3_,… *X_λ__n_*, are the wavelengths at which the correlation of the concentration of the components is maximum (in + or − value). The higher values of those *β* coefficients for each of the parameters studied (Al, Ca, Fe, K, Mg, P, Cr, Cu, Ni, Pb, Zn) together with the wavelength where these coefficients showed their maxima and minima absorption are: Al (*λ*, 1330 and 1556 nm; *β*, 1986.9 and −2060.8); Ca (*λ*, 1500 and 1542 nm; *β*, 21479.9 and 21880.6); Fe (*λ*, 1228 and 1112 nm; *β*, 1481.3 and 1224.0); K (*λ*, 1481.3 and 1224 nm; *β*, −106,030.1 and 85,857.3); Mg (*λ*, 1520 and 1532 nm; *β*, 40,352.5 and −66,976.4); P (*λ*, 1554 and 1968 nm; *β*, 16,557.2 and 19,820.5); Cr (*λ*, 1366 and 1590 nm; *β*, 7.8 and −10.6); Cu (*λ*, 1244 and 1356 nm; *β*, 46.3 and −27.5); Ni (*λ*, 1520 and 1558 nm; *β*, 25.1 and 20.15); Pb (*λ*, 1820 and 1968 nm; *β*, 436.5 and −266.6); Zn (*λ*, 1816 and 1976 nm; *β*, −9692.4 and 6045.0). It is noteworthy that an important number of the mineral elements determined in this work, showed a correlation between the concentration and the absorbance at wavelengths in the 1510–1550 nm interval. According to Shi *et al.*, [49], this region of the spectrum corresponds to the absorbance of two aromatic rings of the basic structure of flavonoids and to the vibration of the 2nd overtone of the carbonyl group of flavonoids. Other mineral elements showed correlations with wavelength values near 1400 and 1900 nm related to the water O-H overtones.

The results showed that it is possible to determine the composition of aluminum, calcium, iron, potassium, magnesium, phosphorus, chromium, copper, nickel, lead and zinc in the ranges indicated in samples of propolis of different origins (from Chile and two Spanish regions) by direct application of a NIR fiber-optic probe on crushed propolis samples without prior treatment or manipulation.

### 3.3. Validation

#### 3.3.1. Internal Validation (Prediction)

**Figure 2:**
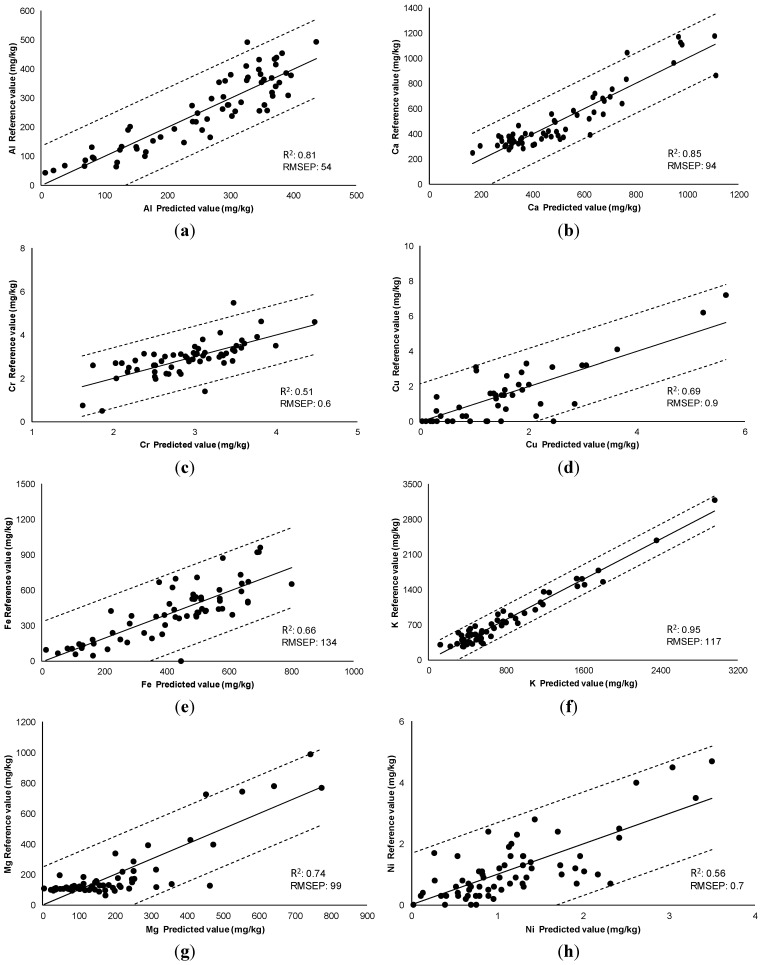

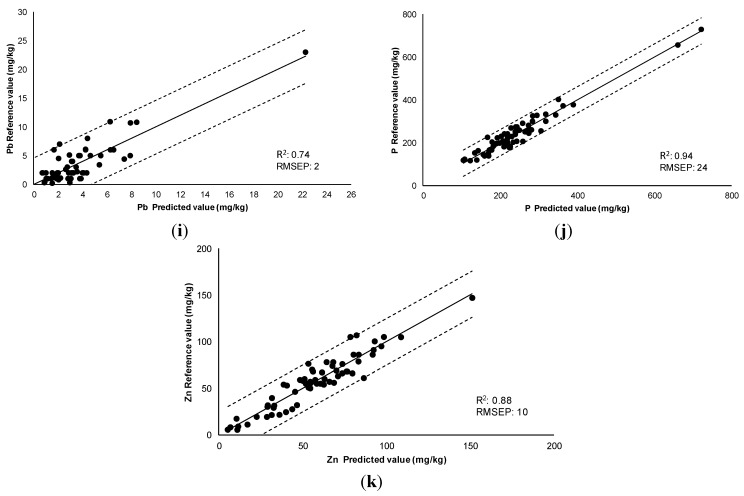
Comparison of reference values (mg/kg) with values predicted by the calibration equations obtained by NIR. R^2^, determination coefficient; RMSEP, root mean square error of prediction. (**a**) Al; (**b**) Ca; (**c**) Cr; (**d**) Cu; (**e**) Fe; (**f**) K; (**g**) Mg; (**h**) Ni; (**i**) Pb; (**j**) P; (**k**) Zn.

Models’ evaluations were performed by cross-validation. In this method, the set of calibration samples is divided into a series of subsets, in our case six. Of these, five were taken for the calibration set and one for the prediction set. The process is repeated as many times as there are sets, so that all pass through the calibration set and the prediction set. Using this process, we validated the models used and checked their prediction capacities. Figure 2 shows the correlation of the values obtained in the laboratory with respect to those predicted by NIR with remote reflectance fibre-optic probe for aluminum, calcium, iron, potassium, magnesium, phosphorus, zinc, chromium, nickel, copper and lead in propolis. The prediction capacity of the model obtained was evaluated with the ratio performance deviation (RPD) [36].This parameter is defined as the relationship between the standard deviation of the chemical method (SD ref) and RMSECV, root mean square error of cross-validation encountered in the NIRS model. Table 3 shows the values obtained for RPD parameter that were comprehended between 3.1 for calcium and 1.0 for nickel. Therefore, NIRS technology presents a capacity for prediction that is interesting for the determination of mineral composition in samples of unknown propolis.

#### 3.3.2. External Validation

**Table 4:** External validation of minerals in propolis by NIR (number of samples: 20).

Constituent	Mean	SD	Est. Min	Est. Max	RMSEP	RMSEP(C)	RPD
Al	239.5	91.8	24.6	370.2	114	113.4	0.8
Ca	946.3	290.6	70.8	1283.0	106.5	116.2	2.5
Fe	392.9	164.7	97,3	745.5	164.2	168.5	1.0
K	1052.1	572.3	453.4	2503.5	250.3	258.2	2.2
Mg	198.4	157.6	63.7	441.8	157.1	165.3	1.0
P	237.0	83.3	77.4	364.7	48.1	46.1	1.8
Cr	3.1	0.64	1.7	3.9	0.92	0.90	0.7
Cu	1.37	1.4	0.2	4.5	1.5	1.6	0.9
Ni	1.4	0.6	0.1	2.4	1.3	1.3	0.5
Pb	4.4	3.1	1.2	14.0	1.2	1.4	2.2
Zn	55.4	28.6	7.1	128.2	18.3	24.1	1.2

SD, standard deviation; Est. Min: minimum value estimated by the model developed; Est Max: Maximum value estimated by the model developed; RMSEP, root mean square error of prediction; RMSEP(C), root mean square error of prediction corrected with bias; RPD, ratio of performance to deviation.

The proposed NIR method was verified by applying the developed chemometric model to 20 new samples of different compositions (called external validation set samples). The recording of the spectra in triplicate and the average spectra was considered. Then, calibration equations obtained in this work were applied and predicted values were compared with reference data for aluminum, calcium, chromium, copper, iron, potassium, magnesium, nickel, phosphorus, lead and zinc determined by OES-ICP and ICP-MS spectrometry. Table 4 shows the results of external validation corresponding with the prediction of mineral composition of 20 independent samples.

## 4. Conclusions

NIR methodology with a remote reflectance fibre-optic probe is presented as an effective method of analysis for determining some mineral and toxic trace elements in crushed propolis. This method is applicable to samples with a wide range of contents of aluminum, calcium, iron, potassium, magnesium, phosphorus, zinc, chromium, nickel, copper and lead. This is the first work on the quantification of mineral elements in a propolis matrix with NIR technology. The method is particularly interesting for the prediction of potentially toxic elements such as zinc, copper and lead, since it allows a previous detection in a short time (3 or 4 min). This ensures the safety and quality of propolis for commercialization in national and international markets.
